# Can temperature-dependent changes in myocardial contractility explain why fish only increase heart rate when exposed to acute warming?

**DOI:** 10.1242/jeb.243152

**Published:** 2022-02-23

**Authors:** A. Kurt Gamperl, Alexander L. Thomas, Douglas A. Syme

**Affiliations:** 1 Department of Ocean Sciences, Memorial University of Newfoundland and Labrador, St John's, NL, Canada A1C 5S7; 2 Department of Biological Sciences, University of Calgary, Calgary, AB, Canada T2N 1N4

**Keywords:** Fish, Thermal tolerance, Diastolic filling, Contraction frequency, Stroke volume, Lengthening work

## Abstract

Fish increase heart rate (*f*_H_), not stroke volume (*V*_S_), when acutely warmed as a way to increase cardiac output (*Q*). To assess whether aspects of myocardial function may have some basis in determining temperature-dependent cardiac performance, we measured work and power (shortening, lengthening and net) in isolated segments of steelhead trout (*Oncorhynchus mykiss*) ventricular muscle at the fish's acclimation temperature (14°C), and at 22°C, when subjected to increased rates of contraction (30–105 min^−1^, emulating increased *f*_H_) and strain amplitude (8–14%, mimicking increased *V*_S_). At 22°C, shortening power (indicative of *Q*) increased in proportion to *f*_H_, and the work required to re-lengthen (stretch) the myocardium (fill the heart) was largely independent of *f*_H_. In contrast, the increase in shortening power was less than proportional when strain was augmented, and lengthening work approximately doubled when strain was increased. Thus, the derived relationships between *f*_H_, strain and myocardial shortening power and lengthening work, suggest that increasing *f*_H_ would be preferable as a mechanism to increase *Q* at high temperatures, or in fact may be an unavoidable response given constraints on muscle mechanics as temperatures rise. Interestingly, at 14°C, lengthening work increased substantially at higher *f*_H_, and the duration of lengthening (i.e. diastole) became severely constrained when *f*_H_ was increased. These data suggest that myocardial contraction/twitch kinetics greatly constrain maximal *f*_H_ at cool temperatures, and may underlie observations that fish elevate *V*_S_ to an equal or greater extent than *f*_H_ to meet demands for increased *Q* at lower temperatures.

## Abstract (precis)

**Summary:** Myocardial contraction and twitch kinetics provide mechanistic explanations as to why heart rate, but not stroke volume, increases in fish with temperature, and why maximal heart rate is constrained at cool/cold temperatures.

## INTRODUCTION

Temperature has been described as the ‘ecological master factor’ for fish (Brett, 1965; Farrell et al., 2007) as it affects numerous physiological processes including the functional capacity of the cardiovascular system (Eliasson and Anttila, 2017). When exposed to an acute rise in temperature, fish increase cardiac output (*Q*) in an attempt to meet their increased demands for oxygen (metabolic rate), a response that is mediated almost exclusively by increases in heart rate (*f*_H_). However, increases in *f*_H_ cannot carry on indefinitely, and at a certain point *f*_H_ plateaus before becoming arrhythmic and dropping back to resting levels just prior to the fish's upper critical temperature (Farrell et al., 1996; Farrell, 2009; Gollock et al., 2006; Leeuwis et al., 2021; Steinhausen et al., 2008; Verhille et al., 2013). Research indicates that this cardiac collapse is likely the result of a combination of factors, including a loss of ventricular excitability due to disruptions in cardiomyocyte ionic currents (Haverinen and Vornanen, 2006, 2020), a decrease in the efficiency of mitochondrial oxidative phosphorylation (Christen et al., 2018; Gerber et al., 2020, 2021; Iftikar and Hickey, 2013; Penney et al., 2014) and, potentially, a loss of nervous function (Andreassen et al., 2020). However, why *f*_H_ alone is responsible for temperature-dependent increases in *Q* is not known, especially when experiments where the capacity to increase *f*_H_ is limited by pharmacological agents (e.g. zatebradine) have shown that increases in *Q* can be mediated through stroke volume (*V*_S_) as temperatures rise (Gamperl et al., 2011; Keen and Gamperl, 2012). These data suggest that there are physiological constraints that prevent increases in *V*_S_ as temperature and *f*_H_ increase, that fish preferentially increase *f*_H_ over *V*_S_, or that increases in *f*_H_ are inescapable as a result of some aspect of the direct effect of temperature on the heart.

With regards to whether there is a reason why fish preferentially increase *f*_H_ over *V*_S_ (i.e. whether there is a physiological or mechanical advantage to the heart), the research to date has not provided such evidence. For example, Syme et al. (2013) showed that measures of work (shortening, lengthening and net) from cod (*Gadus morhua*) trabeculae at 20°C responded similarly under conditions of falling oxygen (*P*_O2_ values) whether tested at low *f*_H_ (35 beats min^−1^) and high *V*_S_ (8% strain) versus high *f*_H_ (70 beats min^−1^) and low *V*_S_ (2.2% strain). However, the strain trajectory was not adjusted to match changes in twitch kinetics in that study. Further, Syme et al. (2013) also showed that lengthening work increased greatly when cod myocardium was paced at high rates (from 75 to 115 beats min^−1^, the normal maximum *f*_H_ in this species is 75 beats min^−1^). These data suggest that lengthening work (the work required to stretch the myocardium) may limit cardiac filling at high *f*_H_. The Frank–Starling mechanism is also intimately involved in the regulation of *V*_S_ in fish, and describes how elevated venous return increases end-diastolic volume, and this results in stretching of the myocardium and an increase in contractility, and therefore *V*_S_ (Farrell and Smith, 2017)_._ However, we have very limited knowledge of the relationship between central venous pressure (CVP) and increased temperature, and thus of the possible role that CVP plays with regard to the lack of an increase in *V*_S_ as fish are acutely warmed. This is in part because the contribution of venous pressure (preload) to cardiac filling in fish varies between species (Ho et al., 2002). Further, the effects of temperature on cardiac preload in fish have only been studied in one species, and over a very limited temperature range (Sandblom and Axelsson, 2006). These authors reported no change in CVP in trout (*Oncorhynchus mykiss*) at temperatures from 10 to 16°C, and this may constrain *V*_S_, in particular as the period of cardiac relaxation (diastolic filling) also decreases as the heart beats faster.

As highlighted in a recent Special Issue in *Journal of Experimental Biology* (Predicting the Future: Species Survival in a Changing World), whether organisms have the capacity to compensate for climate change-related impacts requires a mechanistic understanding of the effects of environmental drivers, and how their interactions influence physiological homeostasis (Franklin and Hoppeler, 2021). Thus, we examined how myocardial work and power at relatively cool (14°C) and high (22°C) temperatures are affected by *f*_H_ (contraction rate) and strain amplitude (related to *V*_S_). Because fish increase *f*_H_ preferentially over *V*_S_ when warmed, we hypothesized that increasing *f*_H_ would enhance power to a greater extent than strain (i.e. *V*_S_) at 22°C, and perhaps be advantageous from the perspective of diastolic filling. Importantly, in these experiments, the period of muscle shortening during the strain cycle was adjusted to match the period over which force was generated by the muscle during contraction, with the remainder of the cycle being diastolic lengthening. This refinement in experimental paradigm resulted in the myocardium generating force while shortening, then relaxing at the onset of lengthening, at all temperature/strain/*f*_H_ combinations, as would occur during the cardiac cycle *in vivo*. This is opposed to a symmetrical strain cycle, which is commonly used in work-loop studies, but which does not accurately mimic the function of a beating heart, particularly at high and low *f*_H_.

The results of the present study indicate that: (1) myocardial work (i.e. stroke work) and power rise substantially with increases in both strain amplitude (i.e. *V*_S_) and *f*_H_, but that increases in *V*_S_ may be less effective than elevating *f*_H_ with regard to increasing cardiac power output when fish are acutely warmed to temperatures approaching their critical thermal maximum (CT_max_); (2) the work required to lengthen the muscle (i.e. diastolic filling work) was very sensitive to changes in strain amplitude (and thus *V*_S_), but was almost independent of *f*_H_ at 22°C, and this may assist the heart to fill at high temperatures and *f*_H_; (3) in contrast, there was a substantial increase in lengthening work at 14°C with increased *f*_H_, and this, combined with constraints on the duration of lengthening at high *f*_H_, may limit maximal *f*_H_ at cooler temperatures. Collectively, these data provide considerable, and novel, insights into the temperature dependence of cardiac function, and mechanistic explanations (hypotheses) for why tachycardia is the predominant/sole mechanism by which fish increase *Q* when acutely exposed to elevated temperatures.

## MATERIALS AND METHODS

### Animal husbandry

All procedures were approved by the animal care committees of the Memorial University of Newfoundland and Labrador (MUN) and the University of Calgary, and followed CCAC guidelines. Adult steelhead trout, *Oncorhynchus mykiss* (Walbaum 1792), were sourced from Nova Fish Farms Inc. and initially housed in a circular, 3000 l, tank supplied with aerated 14°C seawater for approximately 2 months in the Dr Joe Brown Aquatic Research Building at the Ocean Sciences Centre (MUN; St John's, NL, Canada). A 12 h light:12 h dark photoperiod was maintained throughout, and the fish were fed a commercial pelleted trout diet 3 times a week at 1.5% of body mass per feeding. Eighty fish were then removed from the 3000 l stock tank, and transferred to a 1200 l tank for a 4 week acclimation period prior to experimentation; conditions in this tank mirrored those described for the stock tank except that the ration was changed to 1% body mass per day.

### Preparation of isolated myocardium

Prior to an experiment, fish (583±39 g mean±s.e.m.) were netted from the tank and euthanized by cerebral percussion, and the ventricle was excised, cut in half along the sagittal plane and then rinsed in ice-cold physiological saline for marine teleosts (Petersen and Gamperl, 2010). Trabeculae from the spongy ventricular myocardium were isolated on a chilled (4°C) stage using a dissecting microscope. Trabecular preparations were selected so that the majority of fibres ran parallel to the long axis of the preparation, and there was minimal branching along the muscle's length. Preparations (*N*=10, each from a different fish) averaged 1.89±0.35 mg wet mass and had a resting length of 4.87±0.37 mm. A segment of 6–0 silk suture was tied around each end of the preparation and used to attach the muscle strip to stainless steel pins on the arm of a servomotor (Model 300C-LR, Aurora Scientific, Aurora, ON, Canada) and a force transducer (Model 404A, Aurora Scientific). The muscle segments were bathed in physiological saline that was bubbled with air, and the temperature was maintained at 14±0.2°C with Peltier thermoelectric modules and a temperature controller (Model TC-24-12, TE Technology, Traverse City, MI, USA). Platinum plate stimulating electrodes, used to activate the muscle, were positioned on both sides of the preparation and connected to an amplifier circuit powered by a wet cell battery that followed a stimulator (Isostim A320, World Precision Instruments, Sarasota, FL, USA) that was in turn controlled by a computer. A 3 ms, supra-maximal, square voltage pulse was used to stimulate the muscle strips. Custom software written using LabView (National Instruments, Austin, TX, USA) controlled a 12-bit analog/digital converter card (PCI MIO 16E 4, National Instruments) that operated the stimulator and servomotor (5 kHz D/A output) and collected force, muscle length (servomotor arm position) and stimulus signals (1 kHz A/D input). Once attached to the apparatus, the length of each preparation was increased systematically until net power output, measured using the method described below at 30 cycles min^−1^, approached maximal and mimicked the operation of a beating heart on the ascending limb of the force–length relationship.

### Measuring work and power

We evaluated the effects of temperature, contraction rate (i.e. *f*_H_) and strain amplitude (i.e. changes in muscle length, simulating that seen with changes in *V*_S_) on work and power output of the ventricular muscle using the work-loop method (e.g. Carnevale et al., 2021; Johnson and Johnston, 1991; Syme, 1993; Syme and Stevens, 1989). The length of the muscle strips was cycled in a sinusoidal trajectory at a rate of 30, 45, 60, 75, 90 and 105 times per minute (analogous to *f*_H_ in beats min^−1^). The order in which the series of contraction rates was tested (low-to-high versus high-to-low) was alternated between each muscle strip. Strain amplitude was set to 8%, 11% and 14% peak-to-peak (where strain is the change in length relative to the resting length, as a percentage). A strain of 8% is the muscle strain measured at resting stroke volume (*V*_S_=0.4 ml g^−1^ ventricle) in a beating steelhead trout heart (Carnevale, 2019), similar to the value of 9% measured in resting cod hearts (Syme et al., 2013), and 11% and 14% strain bracket values estimated at maximum *V*_S_ in trout (1.3 ml g^−1^ ventricle, ∼12% strain assuming that the ventricle is a sphere) (Carnevale, 2019). The largest strain of 14% also ensured that all strips were operating at, or near, maximum strain as a result of the inherent variability between fish and the varying orientation of trabeculae *in vivo* (Sanchez-Quintana et al., 1995). Experiments were performed at 14 and 22°C; however, a contraction rate of 105 min^−1^ was not used at 14°C as the strips became refractory and could not follow this contraction rate at this temperature. The 14°C temperature was always tested first, followed by 22°C, because higher temperatures tend to be more stressful on muscle. However, to assess the stability of the preparations over the time course of the experiment, work was measured at a reference strain of 11% and 30 beats min^−1^ at the beginning of the experiment at 14°C, at 22°C about mid-way through the experiment, and again at the end of the experiment when preparations were returned to 14°C.

**Fig. 1.**
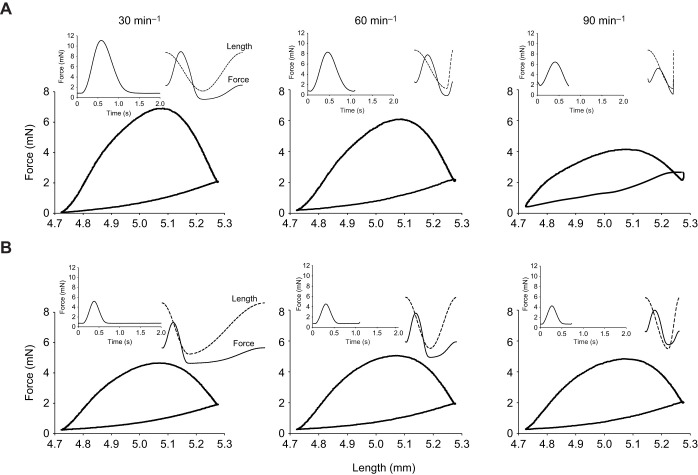
**Example work loops from the spongy ventricular myocardium of steelhead trout.** Trabeculae were paced at (from left to right) 30, 60 and 90 contractions per minute at 14°C (A) and 22°C (B), all at a strain of 11%. Insets in the upper left of each panel show isometric twitch force at the attendant combination of temperature and contraction rate. These were used for calculation of the strain trajectory imposed during the work loops. Insets in the upper right of each panel are the imposed strain trajectory (i.e. muscle length, dashed line) and force produced by the muscle (solid line) during the work loop shown. For the example shown, resting muscle length was 5.0 mm and the mass of the muscle preparation was 1.89 mg.

Importantly, at every combination of contraction rate and temperature, the period of muscle shortening during the strain cycle was adjusted to match the period over which force was generated by the muscle during contraction: the balance of the strain cycle composed of muscle lengthening. As such, the strain trajectory was an asymmetrical sine pattern which resulted in the muscle generating force while shortening and then relaxing at the onset of lengthening (see Fig. 1), as would occur during the cardiac cycle *in vivo*. To calculate the relative proportion of the strain cycle that comprised muscle shortening, isometric contractions were recorded for each muscle strip at each combination of contraction rate and temperature, and the period over which the muscle generated force was measured as the time from stimulation until force fell to 10% of maximum. The proportion of the strain cycle that comprised shortening at each contraction rate and temperature was then set to match the period of force production, calculated as: period of force production/total period of the cycle. The point that the stimulus was applied during the strain cycle was set to coincide with the point at which the muscle strip was at its maximum length, just prior to shortening. This resulted in elastic recoil of the myocardium initiating muscle shortening, but active force being produced primarily during muscle shortening, and avoided variability in stimulus timing as a complicating factor in data interpretation. This timing was expressed as a fraction of the total cycle period and calculated as: (total period of one cycle−period of shortening)/(2×total period of one cycle).

At each combination of strain, temperature and contraction rate, the muscle strip performed 30 consecutive cycles of work, with measurements taken from the last cycle in the series (i.e. where force and work had stabilized). Records of muscle force and length were subjected to a 10-point median filter before analysis to remove any small noise artifacts (Syme et al., 2013). Work was calculated as the sum of the products of length change and average force produced by the muscle over each collection interval. Shortening work was the sum over the shortening portion of the strain cycle, i.e. that associated with ejection of blood in a beating heart. Lengthening work, the work required to extend the ventricular muscle and associated with diastolic filling work of a beating heart, was the sum over the lengthening portion of the strain cycle. Net work was calculated as shortening work minus lengthening work. Values of work done per cycle are shown in the Results as they are particularly useful for interpreting the effects of strain amplitude and temperature on the work required to fill the heart (i.e. diastolic filling work) or eject blood (i.e. stroke work) during each cardiac cycle. However, myocardial power (calculated as the product of work per cycle and cycle frequency) is also presented as it is most useful for interpreting the effects of contraction rate and temperature on cardiac power output (which reflects cardiac output, *Q*). Work is expressed as J kg^−1^ of muscle mass, and power as W kg^−1^ of muscle mass. The mass of the strips was measured at the conclusion of each experiment by removing the muscle preparation from the apparatus, trimming any obviously non-viable tissue, blotting it on filter paper to remove surface moisture, and weighing it on a microbalance (Mettler UMT2, Mettler Toledo, Columbus, OH, USA).

### Statistical analyses

**Fig. 2.**
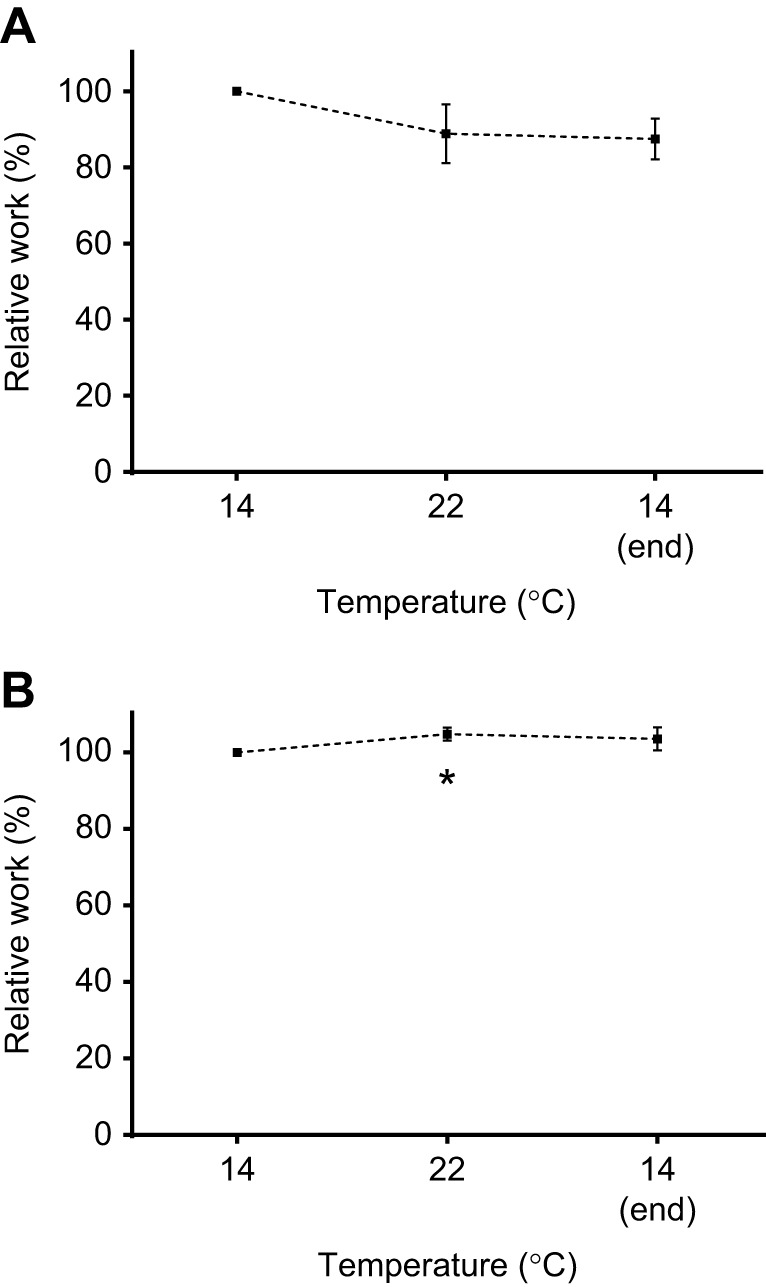
**Work performed by the muscle over the experiment.** Shortening (A) and lengthening (B) work from rainbow trout ventricular trabeculae at 30 contractions min^−1^ and 11% strain amplitude over the duration of the experiment. Data are shown relative to the work done at 14°C during the initial phase of the experiment. Values at 22°C were recorded midway through the experiment. Preparations were then returned to 14°C at the end of the experiment after the measurements at all temperatures, strains and rates were complete, to assess their stability. The asterisk indicates a significant difference (*P*<0.05) as compared with initial values. This statistical analysis was performed on the absolute values of work at each time point. Values are means±s.e.m., *N*=10.

**Fig. 3.**
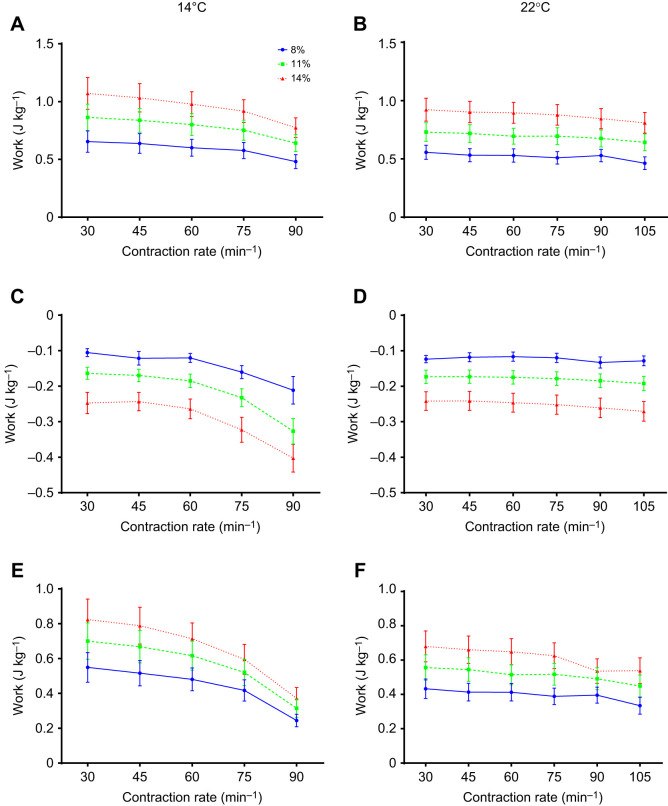
**Effects of contraction rate, strain amplitude and temperature on myocardial work.** Shortening (A,B), lengthening (C,D) and net (E,F) work done per cycle by rainbow trout ventricular trabeculae at different contraction rates and strain amplitudes (8%, 11% and 14%) at 14°C (left) and 22°C (right). There were significant strain and contraction rate effects as summarized in Table 1. Values are means±s.e.m., *N*=10.

**Fig. 4.**
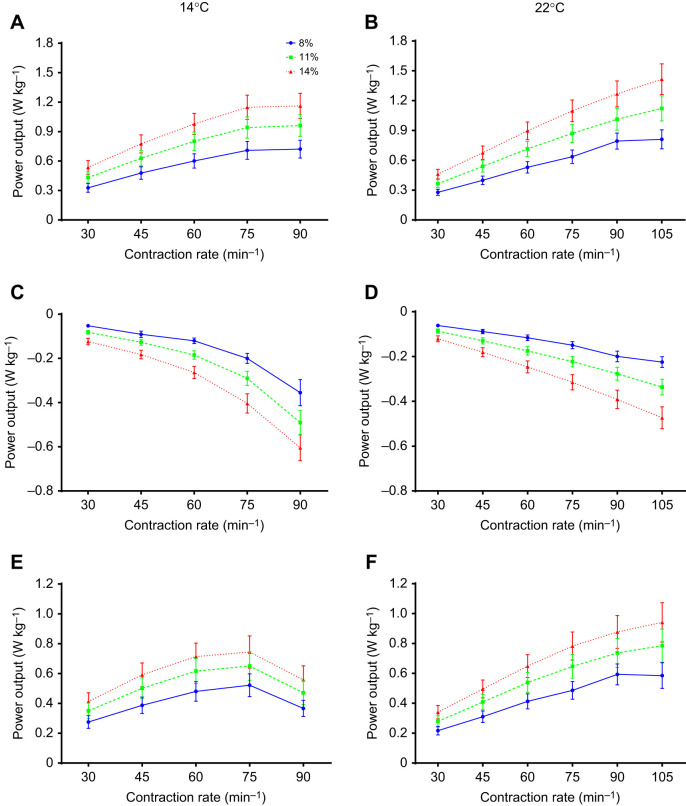
**Effects of contraction rate, strain amplitude and temperature on myocardial power.** Shortening (A,B), lengthening (C,D) and net (E,F) power from rainbow trout ventricular trabeculae at different contraction rates and strain amplitudes (8%, 11% and 14%) at 14°C (left) and 22°C (right). There were significant strain and contraction rate effects as summarized in Table 1. Values are means±s.e.m., *N*=10.

Time-dependent effects on shortening and lengthening work, measured at the beginning, mid-point and end of each experiment at a reference strain of 11% and a contraction rate of 30 min^−1^ (i.e. to test for any loss of contractile performance over the experiment), were examined using one-way repeated measures ANOVA followed by Dunnett's tests (Fig. 2). Split-plot mixed general linear models, and the R statistical package (version 3.22; http://www.R-project.org/), were used to examine the effects of the three controlled variables (contraction rate, strain and temperature) and one random variable (strip) on measures of work and power (Table 1, Figs 3 and 4). Ventricular trabeculae were not controlled for size, which contributed to inherent variability between strips, but was accounted for in the main model by including strip as a random factor.

**Table 1.**
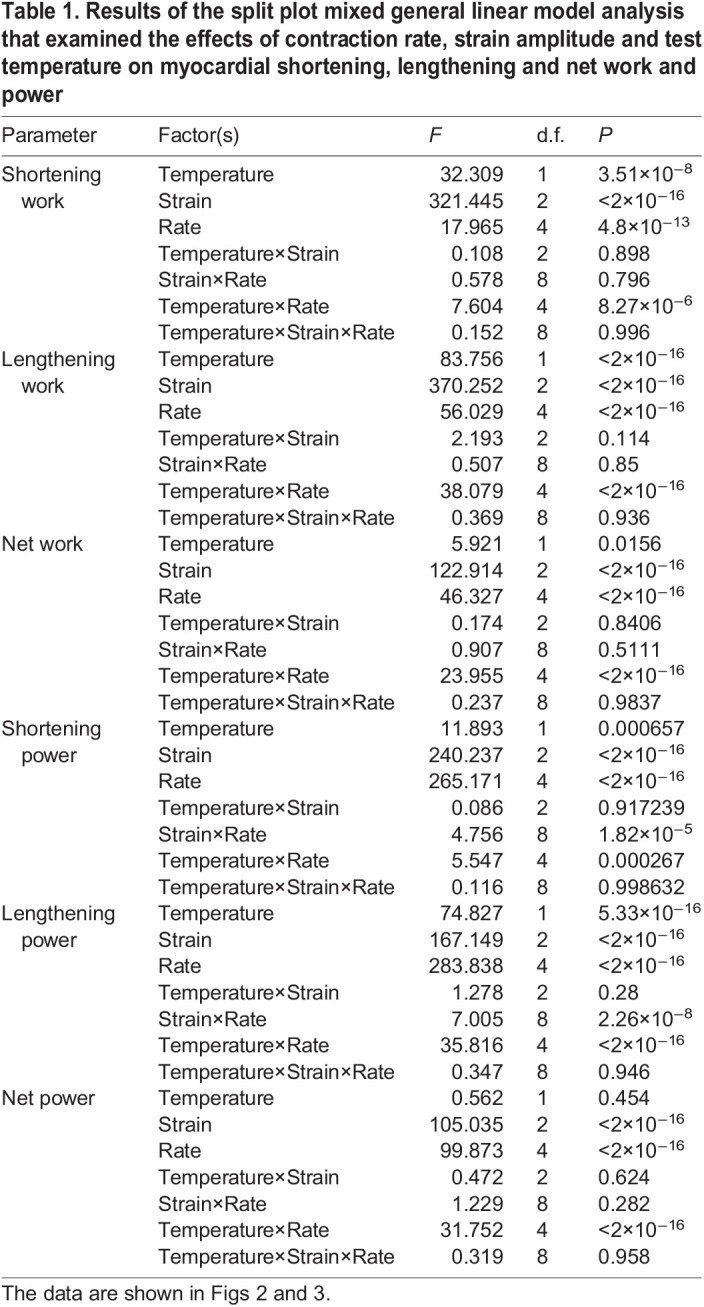
Results of the split plot mixed general linear model analysis that examined the effects of contraction rate, strain amplitude and test temperature on myocardial shortening, lengthening and net work and power

**Fig. 5.**
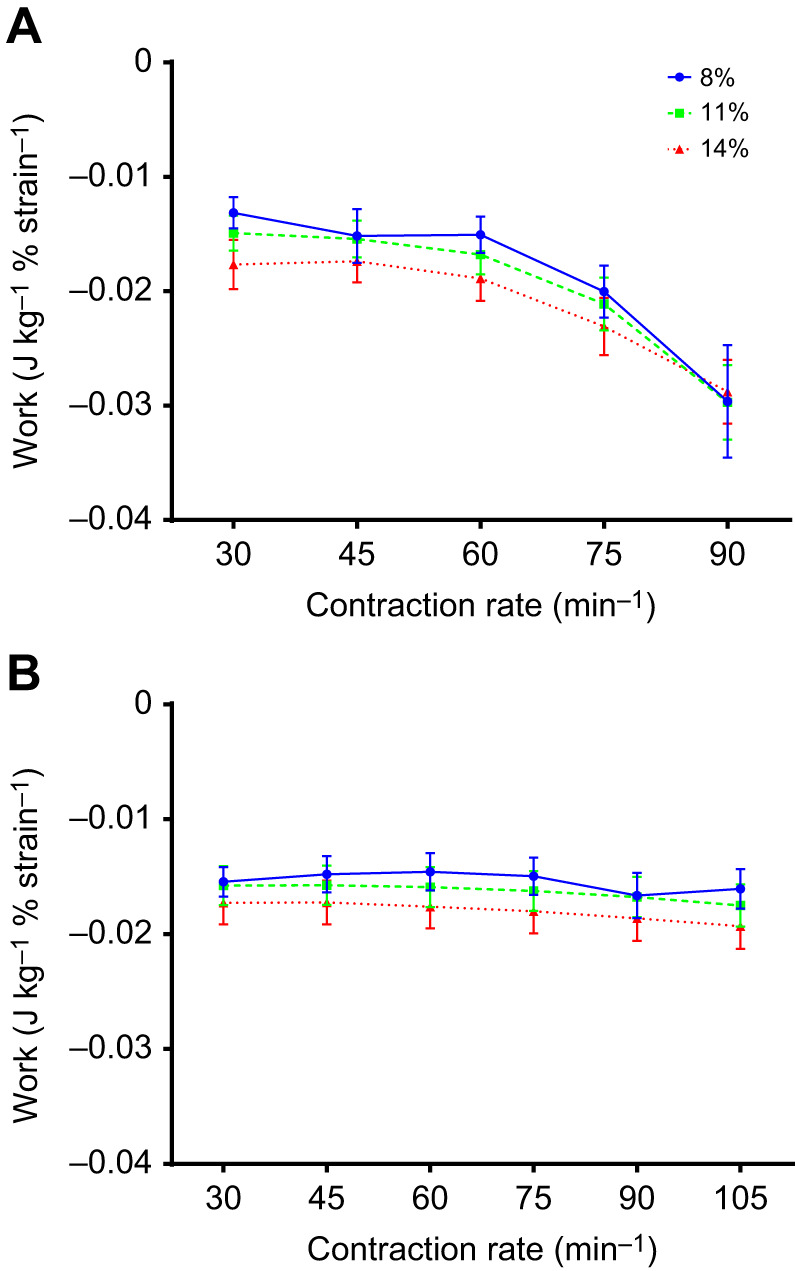
**Temperature-dependent effects on lengthening work.** Lengthening work per unit strain amplitude per cycle from rainbow trout ventricular trabeculae at different contraction rates and strain amplitudes (8%, 11% and 14%) at 14°C (A) and 22°C (B). There were significant strain and contraction rate effects, summarized in Table 2. Values are means±s.e.m., *N*=10.

**Fig. 6.**
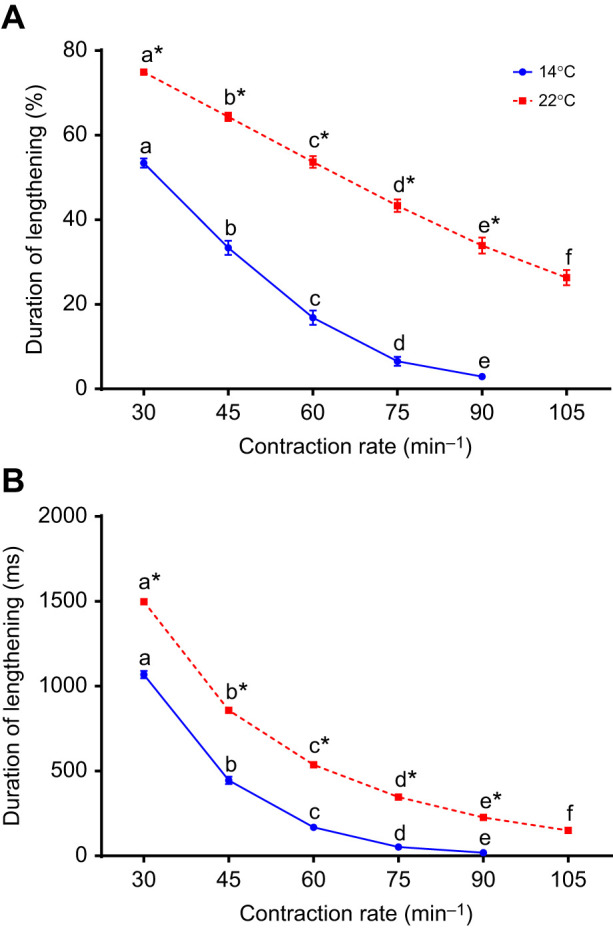
**Duration of the strain cycle attributed to lengthening.** Data are expressed as a percentage of the whole cycle (A) and in milliseconds (B) at different contraction rates and two different temperatures (14 and 22°C) for rainbow trout ventricular trabeculae. Within a temperature, symbols without a letter in common are significantly different (*P*<0.05). Asterisks indicate significant differences between temperatures at a specific contraction rate. Values are means±s.e.m., *N*=10.

**Fig. 7.**
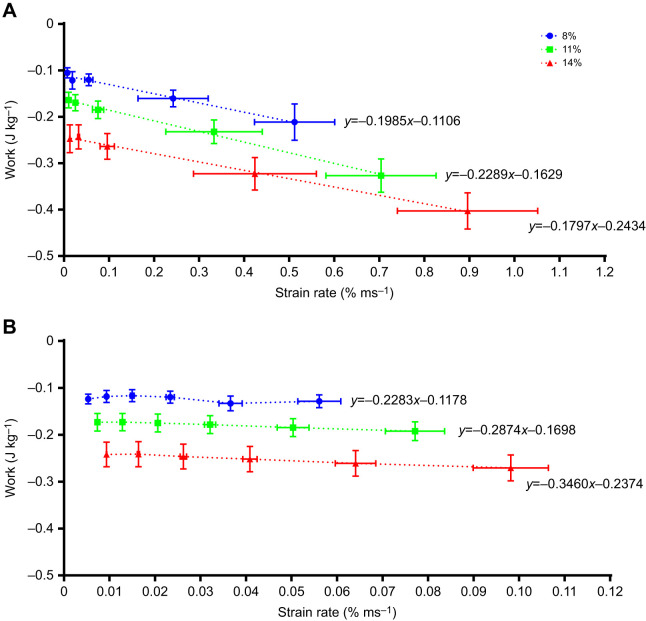
**Effects of lengthening strain rate on lengthening work of rainbow trout ventricular trabeculae.** Strips were tested at different contraction rates and strain amplitudes (8%, 11% and 14%) at 14°C (A) and 22°C (B). Note the order of magnitude difference between the strain rate axes at the two temperatures. The equations are linear regressions based on the individual data points. In A, all the slopes are significant (*P*<0.01), and while the intercepts of the lines are significantly different between strains, the slopes are not different. In B, none of the regressions have significant slopes, but the intercepts are significantly different. Values are means±s.e.m. of data pooled at each contraction rate, *N*=10.

Values of lengthening work done per unit strain amplitude at each temperature (Table 2, Fig. 5) were analysed using a two-way repeated measures ANOVA (with controlled variables contraction rate and strain). This was followed by one-way repeated measures ANOVA and Tukey's HSD tests. Differences in the relationship between lengthening work and strain rate at the three different strain amplitudes (8%, 11%, 14%; Fig. 7) were examined at each temperature (14 and 22°C) using linear regression analysis. This included testing whether the intercepts and slopes of the lines were significantly different at all temperatures. The duration of lengthening per cycle, as affected by temperature and contraction rate (Fig. 6), was examined using a one-way repeated measures ANOVA, followed by: (1) paired *t*-tests between temperatures; and (2) one-way ANOVA followed by Tukey's HSD tests between contraction rates. Finally, specific (*a priori*) comparisons were made of lengthening work and shortening power measured at 14°C at 60 contractions min^−1^ and 8% strain (*in vivo* conditions), as compared with two particular strain/contraction rate combinations at 22°C, using repeated measures ANOVAs followed by Holm–Šidák tests (Table 3). These strain/rate combinations reflected possible *in vivo* conditions at warmer temperatures. The ANOVA, Tukey's HSD tests and *t*-tests were performed using Prism 8 (GraphPad Software, San Diego, CA, USA). *P*<0.05 was used as the level of statistical significance in all analyses, and all values in the text, tables and figures are means±s.e.m.

**Table 2.**
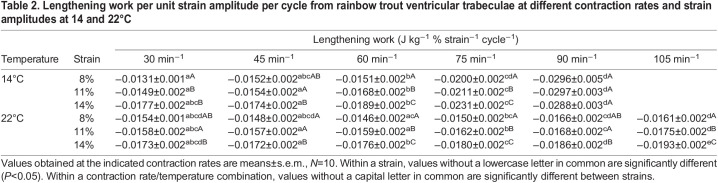
Lengthening work per unit strain amplitude per cycle from rainbow trout ventricular trabeculae at different contraction rates and strain amplitudes at 14 and 22°C

**Table 3.**
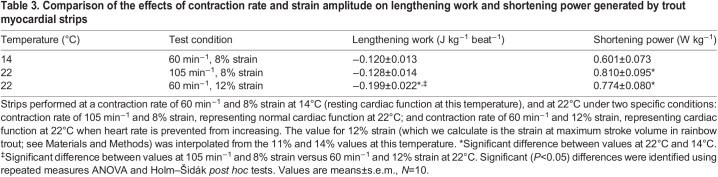
Comparison of the effects of contraction rate and strain amplitude on lengthening work and shortening power generated by trout myocardial strips

## RESULTS

The shortening work performed by myocardial strips (at a reference strain of 11% and 30 contractions min^−1^) at 14°C at the end of the experiment was not significantly different from the work measured at 22°C mid-way through the experiment, or at 14°C at the beginning of the experiment (Fig. 2A). Lengthening work was also not significantly different between the start and end measurements at 14°C, and was only about 4% higher (*P*<0.05) at 22°C (Fig. 2B). The preparations, were thus, very stable over the course of an experiment.

### Effects of strain on work and power

At both temperatures, changes in shortening work done per cycle, and changes in shortening power, were positively but less than proportionally related to changes in strain amplitude (Table 1); shortening work and power were about 33% greater at 11% versus 8% strain, and about 63% greater at 14% versus 8% strain (Figs 3A,B and 4A,B). Lengthening work and power also increased with strain amplitude (Table 1). However, the increase was proportionally greater than the increase in strain amplitude (Figs 3C,D and 4C,D). For example: at 14°C, lengthening work and power were about 150% greater at 14% versus 8% strain at a contraction rate of 30 min^−1^ and about 100% greater at 90 min^−1^; and at 22°C, lengthening work and power were about 100% greater at 14% versus 8% strain across all contraction rates. This disproportionate increase in lengthening work was further examined by plotting work per unit of strain amplitude (Fig. 5, Table 2), where more negative values indicate more work was required for a given amount of lengthening. Clearly, there was a significant increase in the amount of work required to lengthen the myocardium per unit strain at larger strain amplitudes. Net work and power also had a positive relationship with strain amplitude (Figs 3E,F and 4E,F, Table 1), but the magnitude of the increase was noticeably less at higher contraction rates at 14°C. This reflected the high levels of lengthening work under these conditions, particularly at high strain.

### Effects of contraction rate on work and power

Shortening work done per cycle declined slightly with increased contraction rate at all strain amplitudes, although the extent of the decline was marginally greater at 14 versus 22°C, particularly at higher contraction rates (Fig. 3A,B, Table 1). At 14°C, these changes in work per cycle resulted in an increase in shortening power that was approximately proportional to contraction rate up to ∼75 min^−1^, but then approached a plateau at 90 min^−1^ (Fig. 4A), whereas at 22°C, power continued to increase in approximate proportion to contraction rate up to 105 min^−1^ (Fig. 4B). Conversely, while there was almost no change in lengthening work per cycle as contraction rate increased at 22°C, a large increase in lengthening work was observed in muscle working at 14°C (∼5% versus 60%, respectively; Fig. 3C,D, Table 1). This is highlighted by the approximately 2-fold increase in lengthening work per unit strain with increasing contraction rate at 14°C, but almost no change at 22°C (Fig. 5, Table 2). Lengthening power also increased with contraction rate (Table 1, Fig. 4C,D), but the extent of the increase was greater than the increase in contraction rate, and highly dependent on temperature. For example, at 14°C, lengthening power increased about 6-fold over the 3-fold range of contraction rates (30 to 90 min^−1^), while at 22°C, it increased less than 4-fold.

In combination, these changes in shortening and lengthening work lead to a decrease in net work with increased contraction rate, with the decline considerably greater in muscle working at 14 versus 22°C (Fig. 3E,F, Table 1). As a result, net power at 14°C initially increased with contraction rate, but then attained a maximum at ∼75 min^−1^ and subsequently declined. In contrast, net power at 22°C continued to increase with contraction rate, with little evidence of it approaching a maximum (Fig. 4E,F).

### Lengthening rate, duration and work

In a beating heart, the period of muscle shortening is determined largely by the duration of the cardiac twitch, with diastolic lengthening comprising the remainder of the cycle – a situation that we mimicked in the present study (Fig. 1). This period of lengthening, and in turn the rate of lengthening, is thus constrained by both the duration of the twitch and *f*_H_ (which sets the total period of time available for shortening and lengthening). Therefore, we examined how contraction rate (*f*_H_) affected the time available for muscle lengthening (equivalent to the duration of diastolic filling) (Fig. 6), and assessed the combined effects of strain amplitude and contraction rate on the rate of muscle lengthening (strain rate) and lengthening work (Fig. 7). The time available for muscle lengthening, expressed either as a percentage of the total cardiac cycle or as time (in ms), decreased as contraction rate increased (Fig. 6). Of note, at 14°C, the lengthening period became extremely brief at high contraction rates, only several per cent of the entire cycle and a few milliseconds in duration, while at 22°C, the lengthening period was significantly longer than that at 14°C and remained a substantial portion of the entire cycle.

As a result of the decreased time available for lengthening with increased contraction rates, the rate of muscle lengthening (i.e. lengthening strain rate) also increased with contraction rate and strain amplitude because the muscle was lengthened a greater amount in the same period of time (Fig. 7). Conspicuously, the lengthening (strain) rates at 14°C (0.1–1% ms^−1^) were about 10-fold greater than at 22°C (0.01–0.1% ms^−1^) (compare *x*-axes in Fig. 7A,B). This resulted in the effect of strain rate on lengthening work being decidedly (and significantly) negative at 14°C at all strain values (i.e. lengthening work approximately doubled). In contrast, when the muscle was working at 22°C, the relatively low rates of lengthening, and the relatively small increase in strain rate that occurred as contraction rate increased from 30 to 105 min^−1^, resulted in no effect of contraction rate on lengthening work.

## DISCUSSION

Fish increase *f*_H_ almost exclusively when exposed to acute increases in temperature, while *V*_S_ remains unchanged, even to the point of fatigue, regardless of whether the fish is resting or swimming (Farrell, 2009; Gollock et al., 2006; Joaquim et al., 2004; Leeuwis et al., 2019; Motyka et al., 2017; Steinhausen et al., 2008). In support of this observation, we report that while the increase in shortening power for a given relative increase in *f*_H_ at 22°C is only slightly greater than for the same relative increase in *V*_S_, the relationships between strain, *V*_S_, *f*_H_, *Q* and myocardial power suggest that increases in strain alone would likely be inadequate to increase myocardial power and *Q*, while changes in *f*_H_ would. Further, at the warmer temperature (22°C), we found that the increase in work required to lengthen the myocardium (diastolic filling) was considerably greater when increasing *V*_S_ (strain) versus *f*_H_. Thus, from a mechanical perspective, it would appear that increasing *f*_H_ is a preferable strategy over increasing *V*_S_ at warm temperatures. In contrast, at cooler temperatures, even though the myocardium itself has the capacity to increase power until high rates of contraction are attained, the time available for cardiac filling/muscle lengthening quickly becomes limiting as *f*_H_ rises, and so diastolic filling time and increased lengthening work greatly impair the ability of the working heart to increase *f*_H_. Thus, *V*_S_ can, and does, increase to promote increased *Q* when *f*_H_ remains low at cooler environmental/test temperatures (Steinhausen et al., 2008) or during warming with pharmacological blockade (Keen and Gamperl, 2012). While these results are based on measures from spongy trabecular muscle, and thus the effects of specific combinations of contraction rate and strain cannot necessarily be conferred to the compact layer, the twitch kinetics of the two layers differ by only about 10% (Roberts et al., 2021). This suggests that the same patterns of effect should occur in both layers, and broader conclusions regarding the impact of *f*_H_ versus *V*_S_ will apply to the whole heart regardless of tissue type.

### Strain, contraction rate, myocardial power and cardiac output

When the vectors of muscle force and length change are parallel, as they are in the experimental apparatus/conditions used in this study, work done by or on muscle is the product of force and the change in length (strain). Thus, as a first approximation, work output should be directly proportional to strain amplitude, and deviations from proportionality suggest additional impacts on the force produced by the muscle. This has important implications for the effectiveness of altering work or power through changes in strain amplitude (i.e. *V*_S_). Changes in shortening work and power were somewhat less than proportional to changes in strain amplitude (e.g. ∼33% increase in work with a 38% increase in strain from 8% to 11%, and ∼63% increase in work with a 75% increase in strain from 8% to 14%), such that changes in work or power averaged only about 86% of changes in strain (Figs 3A,B, 4A,B). This indicates that force was depressed by increased strain amplitude, and resulted in less work done than anticipated from the increase in strain. This is a common observation, as increased strain results in increased velocity of shortening, which eventually limits work output (Altringham and Johnston, 1990; Johnson and Johnston, 1991; Syme and Stevens, 1989). As a consequence, changes in stroke work in a working heart (i.e. pressure–volume work) during each cardiac cycle would be proportionally less than the increase in strain amplitude (i.e. those associated with changes in *V*_S_).

Alternatively, *f*_H_ could be increased to meet the fish's demands for *Q* and oxygen delivery. Effects of contraction rate (*f*_H_) on *Q* are best considered by assessing power output (i.e. shortening power). At 14°C, shortening power first increased, but then approached or attained a plateau at higher rates of contraction (Fig. 4A), whereas at 22°C, power increased approximately in proportion to contraction rate up to the highest rates measured, and changes in power averaged about 93% of the change in contraction rate (Fig. 4B). This is somewhat higher than the gain in power attained by increasing strain noted above, suggesting that increasing *f*_H_ might be more effective than increasing strain (*V*_S_) to enhance myocardial power output when faced with higher temperatures.

In terms of implications for increasing *Q*, the myocardium must increase power output to at least the same extent as the increase in *Q*, otherwise stroke work and systemic pressure will be compromised. *Q* is directly proportional to *f*_H_, as was myocardial shortening power output for the most part in these experiments (Fig. 4). Thus, increasing *f*_H_ as a means to increase *Q* would provide a good match between the power required to pump blood throughout the fish's circulation and that generated by the myocardium, particularly at 22°C, where power was approximately proportional to *f*_H_ even at maximal *f*_H_. In contrast, relationships between myocardial strain and *V*_S_ (and thus *Q*) are not linear and dependent on heart volume (reviewed by Bijnens et al., 2012). Based on the approach of Syme et al. (2013), Carnevale (2019) measured myocardial strain in steelhead trout at rest (8% at a *V*_S_ of 0.4 ml kg^−1^) and estimated strain at maximal *Q* (12% at a *V*_S_ of 1.3 ml kg^−1^). Using these metrics, a 1.5-fold increase in strain would result in a 3.3-fold increase in *V*_S_ (from 0.4 to 1.3 ml kg^−1^), and thus even if myocardial power was proportional to strain, the increase in power would be substantially less than the increase in *V*_S_ (and *Q*). However, myocardial power was found to be less than proportional to changes in strain in this study, which would further exacerbate this disparity. Hence, the myocardium would clearly be challenged to generate enough power if strain (i.e. *V*_S_) was the sole mechanism available to increase *Q*; inotropy or other aspects of cardiac function would need to change as well. Thus, based on our analysis of muscle mechanics, increases in strain amplitude would appear to be less effective than tachycardia at increasing power output and *Q* at warm temperatures.

The work required to fill the ventricle (lengthen the ventricular myocardium) must also be considered in assessing the effects of temperature on *f*_H_ versus *V*_S_. Lengthening work and power increased considerably more than the change in strain amplitude (i.e. 100–150% increase in work with a 38–75% increase in strain) (Figs 3C,D, 4C,D and 5, Table 2), and this indicates that there is enhanced resistance to muscle lengthening with increased strain. This is likely a result of increased rates of stretch at higher strain amplitudes (Fig. 7), which result in increased resistance to stretch through viscous resistance (Syme, 1990), and through residual cross-bridge activity (particularly if the myocardium is not fully relaxed at the onset of lengthening) (Katz, 1939). Of relevance to *in vivo* cardiac function, diastolic filling (lengthening) work would be predicted to rise disproportionally to the increase in muscle strain amplitude, and hence *V*_S_. In contrast, lengthening power was simply proportional to contraction rate at 22°C (Fig. 4D), as evident by the lack of a change in lengthening work per cycle as the myocardium was paced at higher rates (Fig. 3D), and was much less than the considerable (and disproportionate) increase that occurred when increasing strain. This is likely the result of contraction rate having a very small effect on rates of lengthening at warm temperatures (Fig. 7B), and the fact that there was considerable time available for diastolic filling/lengthening regardless of contraction rate when warm (Fig. 1B, Fig. 6), while the effects of strain are always directly proportional regardless of temperature. Hence, changes in *f*_H_ at warm temperatures have only limited effects on lengthening work, while the impact of strain is large (Fig. 3D). Thus, again, increased strain (and thus *V*_S_) appears to be disadvantageous as a mechanism to increase *Q* from the perspective of diastolic filling work.

To further test the prediction that increasing *f*_H_ might be more advantageous with regard to myocardial performance than increasing strain (i.e. *V*_S_) when temperature is increased, we compared: (1) measures of myocardial shortening power and lengthening work under conditions estimated to mimic what occurs *in vivo* in trout at rest at 14°C (8% strain and *f*_H_ of 60 beats min^−1^) with (2) measures when temperature is increased from 14 to 22°C (i.e. *f*_H_ increasing from 60 to 105 beats min^−1^ while strain remains unchanged at 8%) (Keen and Gamperl, 2012; Motyka et al., 2017); and with (3) what would occur under conditions where *V*_S_ increases to its maximum (1.3 ml kg^−1^, 12% strain amplitude) but *f*_H_ remains unchanged at 60 beats min^−1^ [similar to what occurs when fish are exercised at cool temperatures (Steinhausen et al., 2008), or when *f*_H_ is pharmacologically prevented from increasing when temperature is raised (Keen and Gamperl, 2012)]. There was a significant, and similar, increase in shortening power (which would support increased *Q*) upon warming to 22°C when increasing either contraction rate or strain amplitude (Table 3). In contrast, while there was no significant change in lengthening work on warming to 22°C when *f*_H_ was increased and strain remained constant, there was a substantial and significant (∼65%) increase in lengthening work when strain was increased and *f*_H_ remained low (Table 3). This supports the conclusion that increased *f*_H_ may be preferable, mechanically, to promote diastolic filling of the heart and support increased *Q* at warmer temperatures. However, we note that the specific values of strain employed in this comparison were estimates based on the assumption that the heart is a sphere, and thus may not be completely accurate. Because lengthening work is quite sensitive to strain (see Fig. 3C,D), it would be important to confirm these assumptions with empirical measures of relationships between changes in stroke volume and strain in trout hearts before we can be confident in this particular comparison.

Other factors may also contribute to the observed relationships between temperature, *f*_H_ and *V*_S_. Increased *f*_H_ with temperature has been suggested to be an obligatory response, and likely associated with the effects of temperature on the cardiac pacemaker (e.g. Steinhausen et al., 2008). The increase in *f*_H_, and resultant increase in *Q*, may actually preclude the need for increased *V*_S_ if the increase in *Q* is adequate to satisfy metabolic needs. In fact, this would be the anticipated outcome if *f*_H_ and metabolic rate exhibit a similar *Q*_10_. Hence, the lack of an increase in *V*_S_ with temperature may simply reflect the lack of need for any compensation in addition to that achieved by increased *f*_H_. Alternatively, the lack of an increase in *V*_S_ with temperature could also have a basis in changes in plasma pH, potassium and *P*_O2_ with warming that limit inotropy, and thus the capacity to elevate *V*_S_ [see Steinhausen et al. (2008) for a discussion]. However, when Sockeye salmon are exercised at 15°C, *f*_H_ increases by only about ∼20%, while *V*_S_ increases by ∼80% (Steinhausen et al., 2008), and in steelhead trout, when *f*_H_ is pharmacologically blocked from increasing, *V*_S_ increases with temperature to the extent that *Q* matches what occurs when *f*_H_ is allowed to increase (Keen and Gamperl, 2012). These observations suggest that *V*_S_ can increase, and thus there must be additional physiological constraints and/or mechanistic explanations as to why increases in *f*_H_ versus *V*_S_ are used (favoured) when fish are exposed to various abiotic and/or biotic challenges. Neural and humoral influences (e.g. the release of catecholamines and the stimulation of cardiac β-adrenoreceptors) on the heart would also impact relationships between temperature and the ability of the heart to increase *Q* via *f*_H_ versus *V*_S_. Their effects would ultimately be to modify the same mechanical mechanisms discussed here, including the availability of power for cardiac ejection, the work associated with diastolic filling, and the time available for diastolic filling as described below. In fact, Ask et al. (1981) showed that adrenergic stimulation is particularly relevant with regards to adjusting trout cardiac performance at lower temperatures, and Keen et al. (1993) showed that *in situ* hearts from trout acclimated to 8°C were approximately 10-fold more sensitive to adrenaline than were hearts from fish acclimated to 18°C. If a similar temperature-dependent effect is observed in fish acutely exposed to increased temperature (e.g. over several hours), this decrease in inotropic support could also partially explain why *V*_S_ does not increase *in vivo* when fish are exposed to rising temperatures.

### Duration of diastole

The duration of the cardiac twitch is dependent on *f*_H_ (e.g. Shiels and Farrell, 1997), but did not decrease in direct proportion to the increase in *f*_H_ (Fig. 1). Thus, the duration of the cardiac cycle that comprised shortening became an increasingly larger fraction of the cycle period, while the duration of lengthening decreased (Figs 1 and 6). As a consequence, at 14°C, where the duration of the twitch was relatively long, there was very little time available for lengthening at higher *f*_H_ (Figs 1 and 6). In fact, at higher *f*_H_, the time available for lengthening was unrealistically brief for a functioning heart, and this suggests that there is a constraint on the ability of the trout heart to function at cool temperatures and high *f*_H_. However, at 22°C, the duration of the cardiac twitch was much briefer than that at 14°C (Fig. 1). Thus, the duration of the cardiac cycle that comprised shortening was relatively brief, and this provided a longer time frame for muscle lengthening (Figs 1 and 6), even at the highest contraction rates the heart could sustain. This very short period of time available for lengthening at the cooler temperature (14°C) also had the effect of imposing very high rates of muscle lengthening, as compared with those at 22°C (Fig. 7), to the extent that there was no effect of *f*_H_ on lengthening work at 22°C, but a very large effect at 14°C (Fig. 3). As a result, when warm, there is considerable scope to increase *f*_H_ up to the maximum that the myocardium can sustain, with little impact on lengthening work or diastolic filling time. In contrast, when the fish is at cooler temperatures, one might expect that the limited time available for cardiac filling, and the resultant high amounts of work required to lengthen the myocardium (fill the ventricle), would greatly limit maximal *f*_H_, despite an ability of the myocardium itself to contract at higher rates. Thus, increasing strain (*V*_S_) might be an alternative to elevating *f*_H_ as a means to increase *Q* at cooler temperatures (assuming that filling pressures are sufficient to accommodate an increase in end-diastolic volume). This hypothesis is supported by observations that salmon increase *V*_S_ to a much greater extent than *f*_H_ when exercised at ‘cool’/lower temperatures (Steinhausen et al., 2008), and steelhead trout increase *V*_S_ with increased temperature when *f*_H_ is pharmacologically limited to 60 beats min^−1^ (Keen and Gamperl, 2012). Thus, while fish are capable of increasing *Q* exclusively through increased *f*_H_ when exposed to an acute temperature increase, they can use increases in *V*_S_ to elevate *Q* when *f*_H_ is constrained at lower temperatures or by pharmacological intervention.

A caveat with the ability of the heart to increase *V*_S_ as a means to enhance *Q* is that CVP (which is responsible for 2/3 of cardiac filling in fishes; Farrell and Smith, 2017) would need to increase, or at least remain constant, to support increased *Q* as temperature increases. CVP has only been measured in salmonids from 10 to 16°C (Sandblom and Axelsson, 2006). Clearly, additional measurements related to the effects of altering *V*_S_ and *f*_H_ on cardiac filling are needed before we can fully understand the relationship between changes in temperature and the contributions of *f*_H_ and *V*_S_ to changes in *Q*. For example, we expect that the slowing of *f*_H_ following zatebradine injection (Keen and Gamperl, 2012) and the concomitant increase in filling time and CVP are critical to the capacity of fish treated with this pharmacological agent to increase *V*_S_ when exposed to acute increases in temperature. An additional limitation of interpreting the effects of *f*_H_ and *V*_S_ on the work required to extend the myocardium in this study is that, even though the duration of the lengthening trajectory imposed on the myocardial strips was adjusted to reflect changes in the duration of the twitch at different temperatures and contraction rates, it was still a sinusoidal trajectory. Therefore, it would not reflect phasic filling of the heart if it occurred (e.g. venous filling versus active atrial contraction versus *visa fronte* filling). Rapid phasic filling would likely augment the limitations imposed by increased filling/lengthening work, but active atrial contraction would provide some reprieve from filling limitations, including reduced times required for diastolic filling at cooler temperatures. We are not aware of any studies that would provide further insight into these questions.

### Perspectives and significance

This study provides novel, and important, mechanistic information that addresses the question of why fish only increase *f*_H_ as a means to elevate *Q* when faced with acute increases in temperature to their CT_max_. Further, it provides several testable hypotheses about the role of increases in *V*_S_ versus *f*_H_ in enabling *Q* to meet increased O_2_ requirements in temperate fish species at cold temperatures, and likely other ectotherms where temperature would have similar impacts on myocardial contractile mechanics. However, additional studies/measurements related to the effects of altering *V*_S_ and *f*_H_ on cardiac filling pressure, and on the effects of acute temperature changes on circulating catecholamine levels and cardiac β-adrenergic sensitivity/responsiveness, are needed before we can fully understand the relationship between changes in temperature and how *Q* is modulated. Such information is critical to improving our understanding of how cardiac function and blood O_2_ transport are potentially constrained in fish exposed to heat waves and/or ‘cold shocks’. Both of these environmental perturbations are increasing in frequency and severity with climate change, and can impact fish survival and distribution (Szekeres et al., 2016; Johnson et al., 2018; Frölicher et al., 2018; Cheung and Frölicher, 2020).

## References

[JEB243152C1] Altringham, J. D. and Johnston, I. A. (1990). Scaling effects on muscle function: power output of isolated fish muscle fibres performing oscillatory work. *J. Exp. Biol.* 151, 453-467. 10.1242/jeb.151.1.453 1919410

[JEB243152C2] Andreassen, A. H., Hall, P., Khatibzadeh, P., Jutfelt, F. and Kermen, F. (2020, preprint). Neural dysfunction at the upper thermal limit in the zebrafish. 10.1101/2020.12.28.424529

[JEB243152C3] Ask, J. A., Stene-Larse, G. and Helle, K. B. (1981). Temperature effects on the β-adrenoreceptors of the trout atrium. *J. Comp. Physiol.* 143, 161-168.

[JEB243152C4] Bijnens, B., Cikes, M., Butakoff, C., Sitges, M. and Crispi, F. (2012). Myocardial motion and deformation: what does it tell us and how does it relate to function? *Fetal Diagn. Ther.* 32, 5-16. 10.1159/000335649 22584107

[JEB243152C5] Brett, J. R. (1965). The relation of size to rate of oxygen consumption and sustained swimming speed of Sockeye salmon (*Oncorhynchus nerka*). *J. Fish. Res. Board Can.* 22, 1491-1501. 10.1139/f65-128

[JEB243152C6] Carnevale, C. (2019). The effects of chronic hypoxia and nitric oxide on myocardial contractility in steelhead trout (*Oncorhynchus mykiss*). *MSc Thesis*, Memorial University of Newfoundland, St. John's. https://research.library.mun.ca/14317/1/thesis.pdf.

[JEB243152C7] Carnevale, C., Syme, D. A. and Gamperl, A. K. (2021). Effects of hypoxic acclimation, muscle strain, and contraction frequency on nitric oxide-mediated myocardial performance in steelhead trout (*Oncorhynchus mykiss*). *Am. J. Physiol. Regul. Integr. Comp. Physiol.* 320, R588-R610. 10.1152/ajpregu.00014.2020 33501888

[JEB243152C8] Cheung, W. W. L. and Frölicher, T. L. (2020). Marine heatwaves exacerbate climate change impacts for fisheries in the northeast Pacific. *Sci. Rep.* 10, 6678. 10.1038/s41598-020-63650-z 32317685 PMC7174322

[JEB243152C9] Christen, F., Desrosiers, V., Dupont-Cyr, B. A., Vandenberg, G. W., Le François, N. R., Tardif, J.-C., Dufresne, F., Lamarre, S. G. and Blier, P. U. (2018). Thermal tolerance and thermal sensitivity of heart mitochondria: Mitochondrial integrity and ROS production. *Free Radic. Biol. Med.* 116, 11-18. 10.1016/j.freeradbiomed.2017.12.037 29294390

[JEB243152C10] Eliason, E. J. and Anttila, K. (2017). Temperature and the cardiovascular system. In *Fish Physiology*, Vol. 36B (ed. A. K. Gamperl, T. E. Gillis, A. P. Farrell and C. J. Brauner), pp. 235-297. London, UK: Academic Press.

[JEB243152C11] Farrell, A. P. (2009). Environment, antecedents and climate change: lessons from the study of temperature physiology and river migration of salmonids. *J. Exp. Biol.* 212, 3771-3780. 10.1242/jeb.023671 19915118

[JEB243152C12] Farrell, A. P. and Smith, F. (2017). Cardiac form, function and physiology. In *Fish Physiology*, Vol. 36A (ed. A. K. Gamperl, T. E. Gillis, A. P. Farrell and C. J. Brauner), pp. 155-264. London, UK: Academic Press.

[JEB243152C13] Farrell, A., Gamperl, A., Hicks, J., Shiels, H. and Jain, K. (1996). Maximum cardiac performance of rainbow trout (*Oncorhynchus mykiss*) at temperatures approaching their upper lethal limit. *J. Exp. Biol.* 199, 663-672. 10.1242/jeb.199.3.663 9318401

[JEB243152C14] Farrell, A. P., Axelsson, M., Altimiras, J., Sandblom, E. and Claireaux, G. (2007). Maximum cardiac performance and adrenergic sensitivity of the sea bass *Dicentrarchus labrax* at high temperatures. *J. Exp. Biol.* 210, 1216-1224. 10.1242/jeb.002881 17371920

[JEB243152C15] Franklin, C. E. and Hoppeler, H. A. (2021). Elucidating mechanism is important in forecasting the impact of a changing world on species survival. *J. Exp. Biol.* 224 Suppl. 1, jeb242284. 10.1242/jeb.242284 33627471

[JEB243152C16] Frölicher, T. L., Fischer, E. M. and Gruber, N. (2018). Marine heatwaves under global warming. *Nature* 560, 360-364. 10.1038/s41586-018-0383-9 30111788

[JEB243152C18] Gamperl, A. K., Swafford, B. L. and Rodnick, K. J. (2011). Elevated temperature, per se, does not limit the ability of rainbow trout to increase stroke volume. *J. Therm. Biol.* 36, 7-14. 10.1016/j.jtherbio.2010.08.007

[JEB243152C19] Gerber, L., Clow, K. A., Mark, F. C. and Gamperl, A. K. (2020). Improved mitochondrial function in salmon (*Salmo salar*) following high temperature acclimation suggests that there are cracks in the proverbial ‘ceiling’. *Sci. Rep.* 10, 21636. 10.1038/s41598-020-78519-4 33303856 PMC7729908

[JEB243152C20] Gerber, L., Clow, K. A. and Gamperl, A. K. (2021). Acclimation to warm temperatures has important implications for mitochondrial function in Atlantic salmon (*Salmo salar*). *J. Exp. Biol.* 224, jeb236257. 10.1242/jeb.236257 33288533

[JEB243152C21] Gollock, M. J., Currie, S., Petersen, L. H. and Gamperl, A. K. (2006). Cardiovascular and haematological responses of Atlantic cod (*Gadus morhua*) to acute temperature increase. *J. Exp. Biol.* 209, 2961-2970. 10.1242/jeb.02319 16857880

[JEB243152C22] Haverinen, J. and Vornanen, M. (2006). Significance of Na^+^ current in the excitability of atrial and ventricular myocardium of the fish heart. *J. Exp. Biol.* 209, 549-557. 10.1242/jeb.02044 16424105

[JEB243152C23] Haverinen, J. and Vornanen, M. (2020). Reduced ventricular excitability causes atrioventricular block and depression of heart rate in fish at critically high temperatures. *J. Exp. Biol.* 223, jeb225227. 10.1242/jeb.225227 32434803

[JEB243152C24] Ho, Y.-L., Shau, Y.-W., Tsai, H.-J., Lin, L.-C., Huang, P.-J. and Hsieh, F.-J. (2002). Assessment of zebrafish cardiac performance using Doppler echocardiography and power angiography. *Ultrasound Med. Biol.* 28, 1137-1143. 10.1016/S0301-5629(02)00564-1 12401383

[JEB243152C25] Iftikar, F. I. and Hickey, A. J. R. (2013). Do mitochondria limit hot fish hearts? Understanding the role of mitochondrial function with heat stress in *Notolabrus celidotus*. *PLoS ONE* 8, e64120. 10.1371/journal.pone.0064120 23724026 PMC3665896

[JEB243152C26] Joaquim, N., Wagner, G. N. and Gamperl, A. K. (2004). Cardiac function and critical swimming speed of the winter flounder (*Pleuronectes americanus*) at two temperatures. *Comp. Biochem. Physiol.* 138A, 277-285. 10.1016/j.cbpb.2004.03.016 15313481

[JEB243152C27] Johnson, T. P. and Johnston, I. A. (1991). Power output of fish muscle fibres performing oscillatory work: effects of acute and seasonal temperature change. *J. Exp. Biol.* 157, 409-423. 10.1242/jeb.157.1.409 1919410

[JEB243152C28] Johnson, N. C., Xie, S.-P., Kosaka, Y. and Li, X. (2018). Increasing occurrence of cold and warm extremes during the recent global warming slowdown. *Nat. Commun.* 9, 1724. 10.1038/s41467-018-04040-y 29712890 PMC5928063

[JEB243152C29] Katz, B. (1939). The relation between force and speed in muscular contraction. *J. Physiol.* 96, 45-64. 10.1113/jphysiol.1939.sp003756 16995114 PMC1393840

[JEB243152C30] Keen, A. N. and Gamperl, A. K. (2012). Blood oxygenation and cardiorespiratory function in steelhead trout (*Oncorhynchus mykiss*) challenged with an acute temperature increase and zatebradine-induced bradycardia. *J. Therm. Biol.* 37, 201-210. 10.1016/j.jtherbio.2012.01.002

[JEB243152C31] Keen, J. E., Vianzon, D. M., Farrell, A. P. and Tibbits, G. F. (1993). Thermal acclimation alters both adrenergic sensitivity and adrenoreceptor density in cardiac tissue of rainbow trout. *J. Exp. Biol.* 181, 27-47. 10.1242/jeb.181.1.27

[JEB243152C17] Leeuwis, R. J. H., Nash, G. W., Sandrelli, R. M., Zanuzzo, F. S. and Gamperl, A. K. (2019). The environmental tolerances and metabolic physiology of sablefish (Anoplopoma fimbria). *Comp. Biochem. Physiol.* (Special Aquaculture Issue) 231, 140-148. 10.1016/j.cbpa.2019.02.004 30743060

[JEB243152C32] Leeuwis, R. H. J., Zanuzzo, F. S., Peroni, E. F. C. and Gamperl, A. K. (2021). Research on sablefish (*Anoplopoma fimbria*) suggests that a limited capacity to increase heart function leaves hypoxic fish susceptible to heat waves. *Proc. R. Soc. B* 228. 10.1098/rspb.2020.2340 PMC7944113 33715435

[JEB243152C33] Motyka, R., Norin, T., Petersen, L. H., Huggett, D. B. and Gamperl, A. K. (2017). Long-term hypoxia exposure alters the cardiorespiratory physiology of steelhead trout (*Oncorhynchus mykiss*), but does not affect their upper thermal tolerance. *J. Therm. Biol.* 68, 149-161. 10.1016/j.jtherbio.2016.03.007 28797475

[JEB243152C34] Penney, C. M., Nash, G. W. and Gamperl, A. K. (2014). Cardiorespiratory responses of seawater-acclimated adult Arctic char (*Salvelinus alpinus*) and Atlantic salmon (*Salmo salar*) to an acute temperature increase. *Can. J. Fish, Aqua, Sci.* 71. 10.1139/cjfas-2013-0569

[JEB243152C35] Petersen, L. H. and Gamperl, A. K. (2010). Cod (*Gadus morhua*) cardiorespiratory physiology and hypoxia tolerance following acclimation to low-oxygen conditions. *Physiol. Biochem. Zool.* 84, 18-31. 10.1086/657286 21050128

[JEB243152C36] Roberts, J. C., Carnevale, C., Gamperl, A. K. and Syme, D. A. (2021). Effects of hypoxic acclimation on contractile properties of the spongy and compact ventricular myocardium of steelhead trout (Oncorhynchus mykiss). *J. Comp. Physiol. B.* 191, 99-111. 10.1007/s00360-020-01318-w 33084921

[JEB243152C37] Sanchez-Quintana, D., Garcia-Martinez, V., Climent, V. and Hurle, J. M. (1995). Morphological analysis of the fish heart ventricle: myocardial and connective tissue architecture in teleost species. *Ann. Anat.* 177, 267-274. 10.1016/S0940-9602(11)80198-6 7541184

[JEB243152C38] Sandblom, E. and Axelsson, M. (2006). Adrenergic control of venous capacitance during moderate hypoxia in the rainbow trout (*Oncorhynchus mykiss*): role of neural and circulating catecholamines. *Am. J. Physiol. Regul. Integr. Comp. Physiol.* 291, R711-R718. 10.1152/ajpregu.00893.2005 16741138

[JEB243152C46] Shiels, H. A. and Farrell, A. P. (1997). The effect of temperature and adrenaline on the relative importance of the sarcoplasmic reticulum in contributing calcium to force development in isolated ventricular trabeculae from rainbow trout. *J. Exp. Biol.* 200, 1607-1621. 9319512 10.1242/jeb.200.11.1607

[JEB243152C39] Steinhausen, M. F., Sandblom, E., Eliason, E. J., Verhille, C. and Farrell, A. P. (2008). The effect of acute temperature increases on the cardiorespiratory performance of resting and swimming sockeye salmon (*Oncorhynchus nerka*). *J. Exp. Biol.* 211, 3915-3926. 10.1242/jeb.019281 19043063

[JEB243152C40] Syme, D. A. (1990). Passive viscoelastic work of isolated rat, *Rattus norvegicus*, diaphragm muscle. *J. Physiol.* 424, 301-315. 10.1113/jphysiol.1990.sp018068 2391652 PMC1189814

[JEB243152C41] Syme, D. A. (1993). Influence of extent of muscle shortening and heart rate on work from frog heart trabeculae. *Am. J. Physiol.* 265, R310-R319. 10.1152/ajpregu.1993.265.2.R310 8368384

[JEB243152C42] Syme, D. A. and Stevens, E. D. (1989). Effect of cycle frequency and excursion amplitude on work done by rat diaphragm muscle. *Can. J. Physiol. Pharmacol.* 67, 1294-1299. 10.1139/y89-206 2611725

[JEB243152C43] Syme, D. A., Gamperl, A. K., Nash, G. W. and Rodnick, K. J. (2013). Increased ventricular stiffness and decreased cardiac function in Atlantic cod (*Gadus morhua*) at high temperatures. *Am. J. Physiol. Regul. Integr. Comp. Physiol.* 305, R864-R876. 10.1152/ajpregu.00055.2013 23883672

[JEB243152C44] Szekeres, P., Eliason, E. J., Lapointe, D., Donaldson, M. R., Brownscombe, J. W. and Cooke, S. J. (2016). On the neglected cold side of climate change and what it means to fish. *Clim. Res.* 69, 239-245. 10.3354/cr01404

[JEB243152C45] Verhille, C., Anttila, K. and Farrell, A. P. (2013). A heart to heart on temperature: Impaired temperature tolerance of triploid rainbow trout (*Oncorhynchus mykiss*) due to early onset of cardiac arrhythmia. *Comp. Biochem. Physiol. A. Mol. Integr. Physiol.* 164, 653-657. 10.1016/j.cbpa.2013.01.011 23370292

